# Moderators of a mHealth Intervention for Adolescent Physical Activity: Intervention Refinement Study

**DOI:** 10.2196/59334

**Published:** 2025-12-05

**Authors:** Christopher C Cushing, Adrian Ortega, David A Fedele, Calissa J Leslie-Miller, Jordan A Carlson, Ann M Davis, Joshua M Smyth

**Affiliations:** 1 Department of Clinical Child Psychology and Life Span Institute, University of Kansas, 1000 Sunnyside Avenue, Lawrence, KS, 66045, United States, 1 7858640713; 2 Division of Developmental and Behavioral Health and Center for Children’s Healthy Lifestyle & Nutrition, Children’s Mercy, Kansas City, MO, United States; 3 Department of Pediatrics, University of Missouri-Kansas City School of Medicine, Kansas City, MO, United States; 4 Nemours Children's Health, Jacksonville, FL, United States; 5 Center for Children’s Healthy Lifestyles & Nutrition, Children's Mercy, Kansas City, MO, United States; 6 Department of Pediatrics, Division of Developmental and Behavioral Pediatrics, Center for Children’s Healthy Lifestyles & Nutrition, University of Kansas Medical Center, Kansas City, KS, United States; 7 Department of Psychology, The Ohio State University, Columbus, OH, United States

**Keywords:** mHealth, mobile health, adolescent well-being, physical activity, personalized, adaptive intervention, optimization, adolescent, teen, teenager, youth, exercise, text message, text messaging, nudge, feasibility, acceptability, mood, energy, fatigue

## Abstract

**Background:**

An adaptive text messaging intervention to promote adolescent physical activity has demonstrated feasibility, acceptability, and preliminary efficacy in a recent proof-of-concept study. To inform future intervention development, a secondary analysis of the data examined how physical activity is influenced by mood, environment, and physical feelings of energy and fatigue.

**Objective:**

This study aims to understand how both macro- and microtemporal variables (eg, psychological and environmental variables at both levels) influence the efficacy of a brief mobile health intervention (ie, NUDGE) for physical activity.

**Methods:**

Using a matched control design, we evaluated the effect of daily positive and negative affect, perceptions of the weather, energy, and fatigue as moderators of the effect of the intervention on 21 intervention participants and 21 matched controls.

**Results:**

Consistent with study hypotheses, macrotemporal (levels of the variable on a 3-week timescale) moderators of intervention effectiveness were observed for positive affect (*P*<.001), negative affect (*P*=.03), energy (*P*<.001), fatigue (*P*<.001), and perceived weather barriers (*P*<.001) for moderate-to-vigorous physical activity. These effects were observed more consistently for moderate-to-vigorous physical activity than for sedentary behavior, which was only significant for energy (*P*<.001). No effects for microtemporal variables (at the day level) were observed.

**Conclusions:**

There appears to be an optimization opportunity for mobile health physical activity interventions that can be achieved by personalizing intervention features and content based on approximately monthly assessments of affect, physical feeling states, and perceived weather barriers.

## Introduction

Physical inactivity is a significant public health concern, with recent data showing less than 25% of youth meet the recommended 60 minutes of moderate-to-vigorous physical activity (MVPA) per day [1,2]. Consequences of low MVPA and high sedentary time over the lifespan have significant well-established links to cardiovascular disease, diabetes, poor respiratory health, and cancer [2]. Levels of MVPA decrease as children transition into adolescence, with the majority of individuals failing to meet physical activity guidelines by the age of 13 years [3]. Therefore, interventions targeting increases in MVPA and decreases in sedentary time are critical for adolescents, with long-term benefits that may accrue through adulthood.

Effect sizes for pediatric physical activity interventions are small, producing an increase in objectively measured MVPA that is less than 5 minutes per day on average [4,5]. To improve the efficacy of physical activity interventions, behavioral scientists are leveraging digital technologies to gain insight into key determinants of adolescent physical activity. An innovative perspective involves examining how macrotemporal factors (ie, slow-changing variables over weeks or months) interact with dynamic, microtemporal factors (ie, fast-changing variables over minutes, hours, or days) to influence adolescent physical activity behaviors [6]. Studying physical activity behavior in this manner may increase the understanding of how fast-changing, microtemporal (eg, daily, within-day) and slow-changing, macrotemporal (eg, long-term) variables affect physical activity engagement and could subsequently inform adaptive intervention technologies that are individually tailored to accommodate these microtemporal and macrotemporal variables.

Studies demonstrate that contextual factors, such as the availability of sidewalks and playgrounds, are related to engagement in physical activity [7]. Other macrotemporal variables related to physical activity include person-specific motivational factors, such as self-efficacy for exercise and perceived behavioral control [8-10]. In addition, Cushing et al [11], as well as Dunton et al [12], demonstrated that microtemporal variability in affect (ie, positive and negative affect), physical feeling states (ie, energy and fatigue), and external context (eg, being at home, rainy weather conditions, or time of day) [13] is related to MVPA and sedentary time, and these findings have been extended to adults as well [14]. The field has successfully established broadly that MVPA interventions work; therefore, the critical next phase of intervention optimization is understanding for whom these interventions work and in what context [15].

Framed within the ORBIT (Obesity Relation Behavioral Intervention Trials) model of intervention development [16], we [17] developed and evaluated the NUDGE (Network Underwritten Dynamic Goals Engine) as an unrandomized proof-of-concept study. NUDGE is a mobile health (mHealth) intervention that uses an automated text-message platform to promote adolescent physical activity by prompting users to self-monitor and set daily goals for minutes of MVPA, which was informed by Carver and Scheier’s [18] Cybernetic Control Theory. NUDGE demonstrated feasibility and preliminary efficacy for improving adolescent MVPA (Cohen *d*=0.83) and sedentary time (*d*=0.40). Evaluating macro- and microtemporal variables and their influence on NUDGE could be especially informative and provide insight into individual determinants of physical activity engagement for intervention development.

This study is a secondary analysis of NUDGE to guide future intervention development within the “refine phase” of the ORBIT model. Briefly, this phase uses available data or literature to iteratively make changes to a program before conducting a fully powered randomized controlled trial. The goal is to understand how both macro- and microtemporal variables (eg., psychological and environmental variables at both levels) influence the efficacy of a brief mHealth intervention (ie, NUDGE) for physical activity. We expected that both macro- and microtemporal positive affect and energy would be related to larger intervention effects (ie, more MVPA and less sedentary time), whereas negative affect, fatigue, and perception of poor weather would be related to a diminished intervention effect.

## Methods

### Participants and Procedures

We combined participants from 2 separate studies with identical procedures, ecological momentary assessment (EMA) protocols, and objective monitoring of physical activity. The primary difference between study protocols was that one study involved the delivery of the NUDGE program to the intervention group (detailed below), whereas the other study involved an attention control group. The research team recruited participants in the NUDGE group in 2017 and the attention-control group from 2015 to 2016. Recruitment occurred across all 4 seasons of the year. Recruitment methods included flyers, information letters, and social media postings. We excluded adolescents from participating if they had vision loss or low vision, could not read English, or had a medical condition that would limit their physical activity.

We conducted phone screenings with adolescents 13‐18 years of age and their caregivers to determine their eligibility for the study and scheduled eligible adolescents for a baseline visit. At that visit, we obtained informed consent and assent for caregivers and adolescents, respectively. We provided participants with an accelerometer to wear on their nondominant wrist for the 20-day study period and trained them on how to respond to the EMA surveys via a study smartphone. To maximize adherence to the protocol [19], we allowed participants to select 4 times per day (2 morning and 2 afternoon and evening) to receive EMA surveys, with each survey delivered at least 2 hours apart. Participants in the NUDGE intervention chose an additional time in the evening to receive text messages to set physical activity goals and a time in the morning to receive a goal reminder text message. Participants received up to US $40 for completing study procedures.

#### NUDGE Intervention

As described elsewhere [17], the NUDGE intervention is an automated chatbot. Users are asked to set a daily physical activity goal in minutes of MVPA, which is stored by the system. Next, users are prompted to self-monitor their MVPA at the end of the day and are provided with feedback based on their goal attainment. This process repeats daily for the 20-day study period. A goal is set based on a user’s self-reported exercise from the prior day. Users are allowed to set slightly more or less ambitious goals depending on their preference, but overall, the system encourages increasingly strenuous goals until users achieve 60 minutes of MVPA in a single day.

#### Matched Attention Control Condition

We drew a matched sample of 21 participants from a 100-person dataset of individuals who did not complete the intervention to create the matched attention control. Attention control participants were matched to NUDGE participants on sex, age, and race and ethnicity, with priority given in the order the variables are listed. Participants who did not receive the NUDGE intervention were given the same study experience. They wore an accelerometer on their nondominant wrist, answered EMA surveys at 4 self-selected times per day for 20 days, and completed the same baseline and exit questionnaires. Research staff explained that the study was an assessment of physical activity and psychological variables, and the reimbursement structure was the same as the NUDGE group.

### Measures

#### Demographics

Adolescents completed self-reported measures of demographic variables (eg, sex as a biological variable and race and ethnicity) during the baseline visit.

#### Physical Activity and Sedentary Time

We used wrist-worn ActiGraph GT3X-BT accelerometers (ActiGraph LLC) to measure physical activity across 20 consecutive intervention days. We initialized accelerometers to detect movements at 30 Hz. We processed the accelerometer data in Actigraph Actilife software v6.10.2 and converted raw measurements into 60-second epochs. We removed sleep and nonwear periods using the Sadeh et al [20] and Troiano et al [21] algorithms, respectively. We used the Chandler algorithm [22], a validated algorithm for measuring MVPA in youth using wrist-worn accelerometers, to define cut points for sedentary behavior, light physical activity, and MVPA.

#### Positive and Negative Affect

We measured positive and negative affect 4 times per day via EMA using the Positive and Negative Affect Schedule for Children (PANAS-C; [23,24]). The PANAS-C has strong psychometric properties for describing positive and negative affect and also demonstrates good test-retest reliability and convergent and discriminant validity [24,25]. Previous studies show delivering the PANAS-C on a mobile phone is feasible, reliable, and successful in gathering EMA responses to measure affect in real time [11,12,26,27]. The positive affective domain included the following mood states: joyful, cheerful, happy, lively, and proud. The negative affective domain included the following mood states: miserable, mad, afraid, scared, and sad. Participants rated the extent to which they experienced each mood state on a 5-point Likert scale from 1, “not at all,” to 5, “extremely.” We summed item responses for the positive and negative affective domains for each survey completion. We then mean-centered these values so that all regression weights were relative to no positive or negative affect. Finally, we averaged these values across the day to produce estimates of positive and negative affect for each study day.

#### Energy and Fatigue

Energy and fatigue were measured using questions from the Profile of Mood States (POMS; [28]) 4 times a day via EMA survey. For energy, 3 items with the highest factor loading from the vigor-activity subscale were used (ie, energetic, full of pep, and vigorous). For fatigue, 3 items with the highest factor loading from the fatigue-inertia subscale were used (ie, fatigued, exhausted, and worn out). Participants rated the extent to which they experienced each mood state on a 5-point Likert scale from 1, “not at all,” to 5, “extremely.” We summed item responses for the energy and fatigue scales for each survey completion. We also mean-centered these values so that all regression weights were relative to no energy or fatigue. We then averaged these values across the day to produce daily estimates of energy and fatigue.

#### Perceived Weather Barriers

We additionally measured participants’ perception of weather during EMA surveys with one item 4 times per day: “What is the weather like outside?” In this variable, the intent is to capture high or low perceptions of weather as a barrier and not actual weather conditions. Participants rated their perception of weather as 1, “too hot,” 2, “too cold,” 3, “just right,” 4, “too rainy,” or 5, “too windy.” Our app was limited and only allowed a single response to this prompt (eg, participants could not say it was both too hot and too windy). For this reason, we recoded responses to indicate endorsements of perceived optimal weather and perceived weather barriers (eg, 0=“just right” and 1=all other responses, respectively). Finally, a daily weather barrier score was generated to capture each of the 4 EMA responses by summing these responses across the day, where lower scores represented fewer perceived weather barriers to MVPA. The final daily weather barrier variable ranged from 0 to 4, where 0 represents a response of just right at each EMA survey and 4 represents an endorsement of a perceived weather barrier at each EMA survey*.* Higher scores indicate more cumulative perceived weather barriers within a day.

### Data Analysis Plan

We first divided each hypothesized moderator (eg, positive and negative affect, energy, fatigue, and perceived weather) into between-person and within-person components. The 4 daily assessments were averaged into day-level variables, and then person-mean centering was used to calculate the within-person value [29]. For example, each participant received a personal between-person value of their positive affect, indicating their average level of positive affect during the study, as well as a within-person value of positive affect for each of the 20 days, indicating their daily positive affect relative to their average. Because within-day scores are aggregated at the day level, the within-person effects can be interpreted as day-to-day fluctuations.

Using SAS (SAS Institute) PROC MIXED, we used first-order autoregressive (AR1) multilevel models for the day-level physical activity and sedentary behavior dependent variables. Hypothesized moderators were specified as interactions with group participation (ie, NUDGE intervention vs attention control) on MVPA and sedentary time. All models were estimated using the full information maximum likelihood estimation [29], and separate models were run for each hypothesized moderator. Based on our prior work, we used a random linear model for time at the day level (with the first day of the study coded 0) in the current analyses [17]. For models with significant interactions, we conducted post hoc probing at the mean, as well as ±1 SD above or below the mean of the moderator to determine simple slopes and regions of significance. Each model is conditioned such that regression coefficients can be interpreted as minutes of MVPA or sedentary time per day.

### Ethical Considerations

All study procedures were approved by the local institutional review board at the University of Kansas (STUDY00140405). Informed consent and assent were obtained for data collected in this project and the study team has followed institutional policies for maintaining privacy and confidentiality. Data in the current manuscript are deidentified.

## Results

### Descriptive Statistics

The final sample consists of 42 adolescents aged 13‐18 years (mean 15.14, SD 1.72) in a matched comparison design (ie, NUDGE vs attention control), based on age and sex (n=21 per group). Participants were predominantly female and Caucasian (32/42, 76.2% female and 28/42, 66.7% Caucasian). Detailed sample demographics are shown in Table 1. Over the course of the assessment period, participants in the intervention group engaged in an average of 46 (SD 42) minutes of MVPA and 649 (SD 174) minutes of sedentary time per day. The attention control group obtained an average of 24 (SD 27) minutes of MVPA and 733 (SD 220) minutes of sedentary time per day.

**Table 1.** Adolescent and caregiver demographic characteristics.

Characteristics	Both groups (N=42)	NUDGE group (n=21)	Attention control group (n=21)
Adolescent age, mean (SD)	15.24 (2)	15.14 (2)	15.14 (2)
Adolescent sex, n (%)			
Male	10 (24)	5 (24)	5 (24)
Female	32 (76)	16 (76)	16 (76)
Race and ethnicity, n (%)			
Caucasian	28 (67)	16 (76)	12 (57)
Latino or Latina	4 (10)	2 (10)	2 (10)
African American	3 (7)	2 (10)	1 (5)
Asian	4 (10)	1 (5)	3 (14)
Other	2 (5)	0	2 (10)
Missing	1 (2)	0	1 (5)
Caregiver’s marital status, n (%)			
Married	29 (69)	14 (67)	15 (71)
Divorced	7 (17)	5 (24)	2 (10)
Never married	4 (10)	2 (10)	2 (10)
Separated	2 (5)	0	2 (10)
Approximate family income, n (%)			
≤$60,000	13 (31)	4 (19)	9 (43)
>$60,000	28 (67)	16 (76)	12 (57)
Missing	1 (2)	1 (5)	—^a^

a Not applicable.

### Model Building

#### MVPA Models

##### Overview

An empty model revealed that the intraclass correlation coefficient for MVPA was 0.43, indicating that 43% of the variance was day-level within-person (level 1) variability plus error and 57% was between-person (level 2). As noted above, all final models included a random intercept for the dependent variable and a random linear slope for time coded as day in the study starting at day 0. All models had a significant main effect for group (*P*=.03), and that parameter was included in all final models. All model effects are relations to MVPA at the day level, and β-weights are unstandardized beta coefficients and represent more or less daily activity relative to the control.

##### Positive Affect

**Figure 1.**
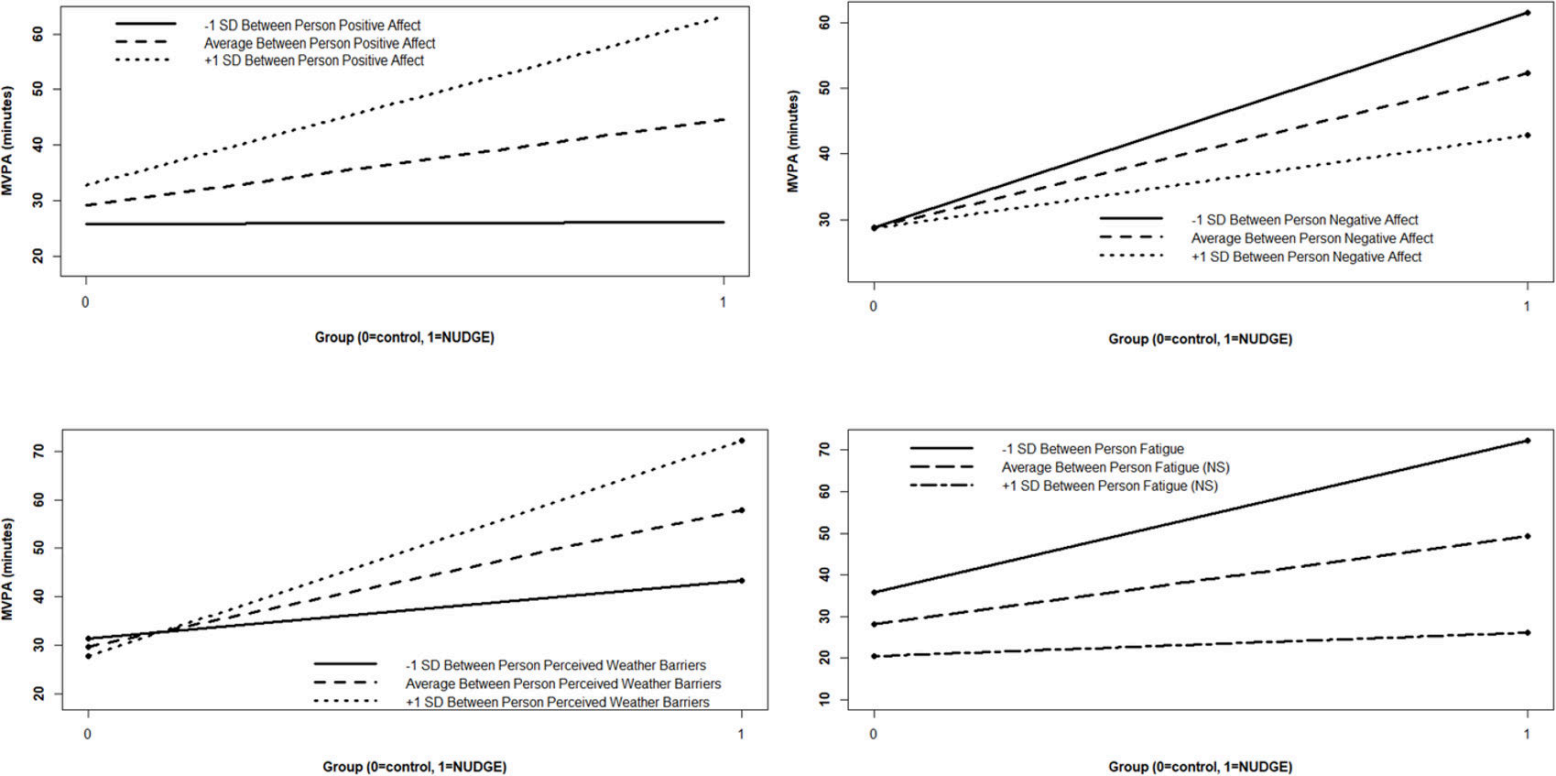
Interaction effects for affect and environmental moderators of the intervention effect. Note: Significant simple slopes, based on post hoc probing, are graphed for interactions where 2 or more levels of the moderator are significant. Nonsignificant slopes are not displayed. The model for fatigue moderating the intervention effect on MVPA did have 1 significant simple slope, but the single line is not displayed. MVPA: moderate-to-vigorous physical activity; NS: nonsignificant; NUDGE: Network Underwritten Dynamic Goals Engine.

There was a significant interaction of between-person positive affect and group (*P*<.001), such that being in the NUDGE intervention (vs control) was related to 30.42 additional minutes/day of MVPA (*P*<.001 from post hoc probing) among individuals with levels of between-person positive affect +1 SD above the mean, but with a nonsignificant simple slope at −1 SD below the mean (See Figure 1A). The interaction between within-person positive affect and group was nonsignificant.

##### Negative Affect

The final model for negative affect indicated a significant interaction between group and between-person negative affect (*P*=.03). Simple slopes indicated that individuals with between-person negative affect +1 SD above the mean obtained 14.08 more minutes/day of MVPA than the control, with those −1 SD below the mean obtaining 32.71 more minutes/day. Generally, participants with less negative affect experienced a greater beneficial effect of the NUDGE on MVPA. The interaction of group and within-person negative affect was nonsignificant.

##### Energy

**Figure 2.**
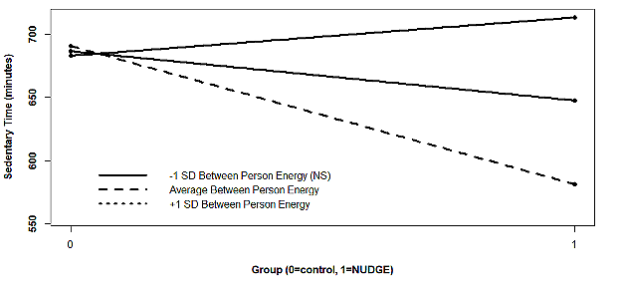
Energy moderating the effect of Network Underwritten Dynamic Goals Engine on sedentary time. Note: Simple slopes, based on post-hoc probing, are graphed for with significant omnibus interactions; NS: nonsignificant; NUDGE: Network Underwritten Dynamic Goals Engine.

The final model for energy predicting MVPA had a significant main effect of group (*P*<.001) and included a significant interaction between group and between-person energy (*P*<.001; Figure 2). However, post hoc probing indicated no significant slopes or regions of significance for this moderator. Within-person energy did not moderate the effect of NUDGE on MVPA.

##### Fatigue

Individuals with lower levels of between-person fatigue (eg, −1 SD below the mean) in the NUDGE condition obtained more MVPA each day relative to their counterparts in the control condition (β=36.54; *P*<.001). Simple slopes were not significant for those with +1 SD above the mean levels of between-person fatigue. The interaction between within-person fatigue and group was not statistically significant.

##### Perceived Weather Barriers

The interaction term was again significant for perceived weather barriers, with those who perceived +1 SD more barriers than the mean obtaining 44.53 more minutes of MVPA each day in the NUDGE group than the control, and those with −1 SD below the mean obtaining only 11.99 additional minutes per day. Interestingly, those with higher levels of between-person who perceived weather barriers in the NUDGE condition obtained more MVPA compared to those with lower perceived weather barriers. Within-person–perceived weather barriers did not moderate the effect of NUDGE on MVPA.

### Sedentary Time Models

#### Overview

An empty model revealed that the Intraclass Correlation Coefficient for sedentary time was 0.37, indicating that 37% of the variance was day-level within-person (level 1) variability plus error and 63% was between-person (level 2). As noted above, all final models included a random intercept for the dependent variable and a random linear slope for time coded as day in the study starting at day 0. All model effects are relations to sedentary time at the day level, and β-weights represent more or less daily activity relative to the control.

#### Positive Affect

The final model for positive affect predicting sedentary time included a significant main effect of group (β=−39.56, *P*=.02) and between-person positive affect (β=−6.39, *P*<.001). Neither between-person nor within-person positive affect moderated the effect of NUDGE on sedentary time.

#### Negative Affect

The final model for negative affect predicting sedentary time included a significant main effect of group (β=−58.05; *P*=.001) and between-person negative affect (B=10.00; *P*=.049). Neither between-person nor within-person negative affect moderated the effect of NUDGE on sedentary time.

#### Energy

The final model for energy predicting sedentary time had a significant main effect of group (*P*<.001) and included a significant interaction between group and between-person energy (*P*<.001). Individuals with +1 SD between-person energy above the mean (β=−109.21, *P*<.001) in the NUDGE group accumulated less sedentary time each day relative to their counterparts in the control condition. The simple slope was not significant at −1 SD below the mean. Within-person energy did not moderate the effect of NUDGE on sedentary time.

#### Fatigue

The final model for fatigue predicting sedentary time had a significant main effect of group (*P*<.001) and included a significant main effect of group (β=−50.33; *P*=.003) and between-person fatigue (β=16.29, *P*<.001). Interactions of between-person and within-person fatigue and the group were not statistically significant.

#### Perceived Weather Barriers

There were no significant main effects of group, between-person or within-person perceived weather barriers, or interactions between these variables predicting sedentary time.

## Discussion

### Principal Findings

This study aimed to determine how macro- and microtemporal variables influence the effects of our mHealth intervention targeting adolescent physical activity. All of the macrotemporal study variables moderated the effect of NUDGE on MVPA, but only macrotemporal energy moderated the effect of NUDGE on sedentary time. With the exception of macrotemporal perceived weather barriers on MVPA, all of our significant interactions were in the hypothesized direction. In other words, adolescents with higher levels of macrotemporal positive affect and energy experienced larger intervention effects (ie, more MVPA and less sedentary time). Similarly, those with lower levels of negative affect and fatigue experienced larger intervention effects. Microtemporal associations with MVPA identified in other studies were not as important in this study as might have been indicated by other associational work [10,12,30]. This study indicates that while microtemporal findings do likely hold potential for personalization, the most immediate opportunity may be at the macrotemporal level. Momentary affect is difficult to capture using passive monitoring; therefore, fortunately, macrotemporal assessment is both easier and more suitable [31,32].

The literature indicates that mHealth interventions produce significant positive health outcomes in youth. The majority of these interventions have used standardized protocols to deliver the same intervention content to users, usually in the form of text messages [33,34]. However, recent developments in mHealth technologies show that mHealth interventions can leverage personalization to promote behavior change [10,35]. In the next iteration of the NUDGE protocol, it may be possible to use baseline information on macrotemporal factors to optimize the intervention for each participant. For instance, participants with low levels of positive affect or high levels of fatigue at baseline may need a preliminary intervention that is more complex than simple text message prompts to set and keep track of their physical activity goals. In both instances, a brief behavioral activation intervention might be warranted to help adolescents acknowledge the importance of setting and meeting goals, even when mood or fatigue are barriers [36].

There are other tailoring opportunities based on how NUDGE interacts with macrotemporal variables assessed at baseline. For example, NUDGE produced a desirable effect for all participants regardless of macrotemporal negative affect or perceived weather. However, it was more effective for those with lower negative affect. For individuals experiencing high negative affect, it may make sense to address this barrier first before enrolling participants in the NUDGE intervention or to use MVPA promotion strategies that are unlikely to be affected by affect (eg, scheduled family exercise). Similarly, the intervention was particularly effective for participants who demonstrated higher than average positive affect. For this group, additional prompts might be wasted contacts that could decrease engagement or lead to habituation [37]. Our results on fatigue suggest that those with higher-than-average fatigue may need a sequencing approach to improve their fatigue before starting on the activity intervention. Finally, the relationship with weather demonstrates that those who perceive more weather barriers derive more benefit from NUDGE, which may indicate the importance of goal-setting and review even in the face of environmental barriers. All of the intervention strategies discussed above fit nicely in phase 2 of the ORBIT model for refining behavioral interventions before proceeding to fully randomized control trials [16].

Within the ORBIT model, NUDGE was tested in a nonrandomized, proof-of-concept study. Initial findings were promising and indicate randomized pilot testing is warranted. However, the ORBIT model encourages behavioral scientists to consider how an intervention can be optimized and refined to deliver more efficient interventions. Rather than making clinical judgments or relying on anecdotal evidence to guide intervention refinement, we can use data from this study to inform the development of future versions of NUDGE. One iteration might use responsive, context-dependent intervention support while another might test tailored decision points for goal setting based on trait-level characteristics (eg, high negative affect and fatigue). This process of using empirical evidence for optimization represents a step forward in the development and refinement of technology-based behavioral interventions [38].

### Limitations and Future Directions

Results from this study should be considered in light of several limitations. Most importantly, participants were not randomized. In addition, the sample was relatively small, participants were predominantly female and Caucasian, and only adolescents from a specific geographic location were included. Future studies should recruit a larger sample of youth to test the generalizability of effects found in this study and provide opportunities for fully powered analyses. In addition, a larger study may be able to identify microtemporal moderators that were not present in this study. While not a major contributor in this study, there is a growing body of literature establishing that microtemporal relationships do affect the health behavior of children and adolescents [30]. Another limitation of this study was our methodology for measuring psychological feeling states. While EMA allowed us to measure daily fluctuations in these states, our protocol instructed adolescents to answer several survey questions 4 times throughout the day for almost 3 weeks. Future studies may benefit from using smart devices (eg, smartwatches) to reduce participant burden by making survey prompts easier to complete and to capture physiological data related to energy and mood (eg, heart rate and skin conductance; [39]).

Findings from this study add further support for the effects of psychological variables and perceived weather barriers on adolescent MVPA and sedentary time. Results highlight the importance of assessing macrotemporal factors (eg, positive and negative affect, energy, fatigue, and perceived weather barriers) in the context of a physical activity intervention for adolescents. While our initial examination of NUDGE showed an overall increase in MVPA, the results from this study indicate that adolescents may respond differently to the NUDGE intervention based on their macrotemporal characteristics, such as positive and negative affect, energy, fatigue, and perceived weather barriers. Future mHealth intervention trials should test the impact of tailored content on adolescents’ MVPA and sedentary time to further refine adaptive physical activity interventions.

### Conclusion

Our secondary data analysis of the NUDGE intervention suggests that patterns of variables, such as affect, energy, fatigue, and weather may moderate the effectiveness of text-message interventions for physical activity. Future iterations of NUDGE may benefit from using tailoring variables to modify the intervention experience of each participant depending on their level of the variables explored in this paper.

## Abbreviations

EMA: ecological momentary assessment
mHealth: mobile health
MVPA: moderate-to-vigorous physical activity
NUDGE: Network Underwritten Dynamic Goals Engine
ORBIT: Obesity Relation Behavioral Intervention Trials
PANAS-C: Positive and Negative Affect Schedule for Children
POMS: Profile of Mood States
